# UV-C Irradiation as a Tool to Reduce Biofilm Growth on Pompeii Wall Paintings

**DOI:** 10.3390/ijerph17228392

**Published:** 2020-11-13

**Authors:** Paola Cennamo, Marta Ebbreo, Giovanni Quarta, Giorgio Trojsi, Alessandro De Rosa, Simona Carfagna, Paolo Caputo, Monica Martelli Castaldi

**Affiliations:** 1 Department of Humanities, University of Naples Suor Orsola Benincasa, 80132 Naples, Italy; martaebbreo@gmail.com (M.E.); giorgio.troisi@hotmail.it (G.T.); m.martelli.c@gmail.com (M.M.C.); 2 ISPC-CNR, c/o Campus Universitario, via Monteroni, 73100 Lecce, Italy; giovanni.quarta@cnr.it; 3 Department of Biology, University of Naples Federico II, 80126 Naples, Italy; alessandro.derosa.proxy@gmail.com (A.D.R.); simcarf@unina.it (S.C.); pacaputo@unina.it (P.C.)

**Keywords:** biofilms, UV-C irradiation, Pompeii, conservation, pigment

## Abstract

This study focuses on the experimentation of a method based on the use of UV-C irradiation to eliminate the biofilms present in a tomb located in the necropolis of Porta Nocera, in Pompeii. For this study, the autotrophic component of the biofilm was isolated in the laboratory, while, contemporarily, the characterization of the composition of the pigments of the frescoes took place on original fragments, which had already detached from the tomb and were examined in situ. These preliminary analyses were necessary for the recreation of test samples in the laboratory, which closely matched the original surfaces. Artificial biofilms were used for experimental exposure to UV-C radiation. The exposure to UV-C radiation was carried out at different distances for a fixed time interval. The effectiveness of the biocidal action was assessed by employing optical microscopy techniques, through a careful visual assessment of the area occupied by the biofilm on the different test samples, using a photographic survey, as well as by means of colorimetric measurements using spectrometric techniques. In order to obtain an additional parameter to evaluate the death rate of microorganism cultures exposed to the UV-C radiation, the concentrations of the photosynthetic pigments were also measured by spectrophotometry. Results showed that biofilms were completely eradicated by radiation, and no change in pigment color was observed.

## 1. Introduction

Biodeterioration represents a significant source of aesthetic degradation for numerous monuments of historical and artistic value. Tombs and crypts are generally considered amongst the so called “confined spaces”, but they constitute peculiar and heterogeneous environments, where the influence of climatic factors frequently exposes part of the underground structures to severe environmental weathering. This allows for the development of ecosystems more typical of “semi-confined structures”, thus producing specific microclimatic conditions suitable for the growth of an abundant microflora. Furthermore, capillary phenomena and infiltrations of water from the ground above cause very high humidity rates. This, together with slight fluctuations in temperature due to the presence of visitors in underground spaces, all contribute to the phenomenon of biodeterioration [1,2]. The growth of biological communities on wall paintings, such as biological patinas, is a common phenomenon, which might cause severe chromatic alterations [2,3,4] but also the deterioration of very fragile decorative surfaces. Over the years, it became apparent that there was a need to control and remove the effects of biodeterioration by using methods alternative to chemical ones. In spite of the effectiveness of patented biocides, such as Biotin R, Rocima 382, and Acticide BAC 50M and their optimal performances in the eradication of a large variety of biodeteriogenic communities, these biocides, unfortunately, leave much to be desired in respect to the structural preservation of fragile surfaces (especially in the case of frequently repeated applications) and to the ecological sustainability of the hosting environment. With respect to the removal of algal biofilms from stone surfaces, UV rays have proven to be an effective ally for disinfection and cleaning; this is because their reduced penetration power does not represent a limit, given the superficial nature of the involved biocoenoses.

In fact, UV-C irradiation has been applied to stone surfaces as early as the 1960s; Dobatenvis used ultraviolet rays to remove the phototrophic microorganisms; later Borderie et al. 2011 [5] showed that UV-C treatment is useful in the elimination of biofilm in caves. Pfendleret al. [6] indicated that in situ UV-C treatment is efficient, faster, cheaper, and more environmentally friendly when compared to chemicals [7]. Recently, a renewed attention towards some cave paintings found inside caverns located in South-Western France has led to the installation of UV-C lamps for conservative purposes [3,5,7]. Not unlike the French caves, Pompeii also represents an ideal condition for the development of autotrophic biofilms, being an archaeological site with variable atmospheric conditions, exposure to artificial lighting and the almost daily presence of visitors. In this regard, the need arises to plan a strategy to reverse this process, so as to be able to guarantee the preservation of these assets. The site selected for our survey and sampling of biodeteriogens, consists of a tomb (ES-07), located in the Porta Nocera necropolis, in the archaeological site of Pompeii, where biodeterioration was evident on many of the pictorial components. A pilot system was developed to expose some test samples with pigments to the biocidal action of UV-C rays, with the aim of simulating the elimination of the artificial biofilm present on the original surfaces.

## 2. Materials and Methods

As previously stated, biofilms were sampled from the frescoed walls of Tomb ES-07, located in the necropolis of Porta Nocera, in the archaeological site of Pompeii.

### 2.1. Chemical-Physical Analysis (XRF)

X-ray fluorescence investigations were carried out for the colorimetric characterization, in order to identify the original pigments present in the decoration. A red, a yellow, and a green pigment were analyzed. X-ray fluorescence measurements were carried out with a portable XRF-Q Assing spectrometer, with a tungsten tube, PiN silicon diode detector with beryllium window, operating conditions 40 KV and 0.5 mA (counting time: 60 s).

### 2.2. Cultivation of Algae

Following the identification of a colony of micro-algae as belonging to Chlorophyceae, two different growth mediums, BG11 and BBM [8,9], were used to elicit exponential growth. The cultures, contained in Erlenmeyer flasks, were maintained in a climatic chamber at 37 °C for 12 days to achieve exponential growth. 

**Figure 1:**
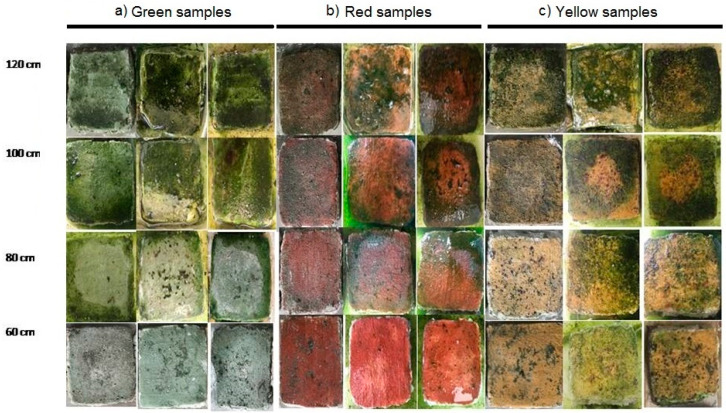
Samples used for inoculation with a mixture of cyanobacteria and green algae at different distances (120, 100, 80, 60 cm). (**a**) green samples; (**b**) red samples; (**c**) yellow samples.

Preparation of the artificial biofilm: A set of test samples was artificially reproduced with the cooperation of the Restoration School of the University of Naples Suor Orsola Benincasa, using the same plaster materials, execution techniques, and pigments used for the original surfaces (Figure 1). A total of 6 test samples, two for each of the three original pigments, were prepared and inoculated with the microalgae cultures, until they were completely covered in biofilm following the procedure of Cennamo et al. [10]. The algal growth on the substrate was carefully monitored in the following days.

### 2.3. UV-C Treatments

The test samples were exposed to the radiation, using two UV-C lamps (Fluorescent Compact G9W Lynx, Sylvania, 2 × 9 W each = 18 W tot, λmax = 254 nm). Five milliliters of algal culture were also extracted from the test samples to be used as control. The irradiations were conducted at 4 different distances (120, 100, 80, 60 cm), for the duration of 8 h a day every other day, for a total duration of twenty-four hours of exposure. 

### 2.4. Light Microscopy and Cell Count

The observation of the samples was carried out using a light microscope (Laborlux 12, Leitz, transmitted light, bright field, 40X zoom). The aliquots of exposed and control algae were subject to direct determination through cell count using a Bürker chamber.

These analyses were performed before and after the treatment, as to be able to discern the variation in cell vitality. 

### 2.5. Absorption of Main Photosynthetic Pigments

Following centrifugation (1′, 5000× *g*), cell pigments were extracted using 2 mL of N,N-dimethylformamide, following the methodology illustrated by Carfagna et al. [11].

Chlorophyll content of cells was estimated spectrophotometrically (Spectrophotometer 7315, Jenway). The absorbance at the wavelengths 647 and 664 nm was used to determine the concentration of total chlorophyll, as well as that of chlorophyll a and b, using the following Equations: Chl Tot = (A647 × 17.9) + (A664 × 8.08); Chla = (A664 × 12.7) − (A647 × 2.79); Chl b = (A647 × 20.78) − (A664 × 4.88).

### 2.6. Colorimetric Analyses

The color measurements were taken by light absorption in diffuse reflection using a Konica Minolta CM700d spectrophotometer. The measurements were executed with a D65 illuminant and under a 10° standard observer. Before starting a measurement, the device was calibrated with a white ceramic disk and a black trap portion. For each sample, 9 measurements were carried out, and for each point, three repetitions were acquired. Then, the instrument automatically calculated a mean value. Reflectance plots in the visible range of wavelengths were acquired. Then, the results of the colorimetric analysis were reported in the framework of a standard three-dimensional colorimetric space using the Minolta SpectraMagic NX (Konika Minolta Inc., Tokyo, Japan) software. The colorimetric data reported in this work were calculated in the CIEL*a*b* 1976 color space [12,13,14]. In this system, L* coordinate is lightness (L* = 0: black and L* = 1: white), a* coordinate represent the green/red component (negative values represent the green component and positive values represent the red component, through grey close to zero), and the b* coordinate refers to the blue/yellow component (negative values representing the blue component and positive values the yellow component, through grey close to zero). The color differences were calculated on average values as (L*a*b*), and E00 color differences were also calculated applying the CIEDE2000 formula [15].

## 3. Results

### 3.1. XRF Analyses of the Pigments

The analysis of the red pigment revealed the presence of calcium and iron, attributable to a red ochre (probably hematite). For the green pigment, the documented presence of iron, silicon, and potassium is to be attributed to a green earth (copper being completely absent) from celadonite (this mineral is well attested in the Somma–Vesuvius volcanic complex). In addition, for the yellow pigment, the presence of only calcium and iron indicates a yellow ochre (probably goethite). As a result, we based the preparation of our test samples on all these findings.

### 3.2. Effect of UV-C on Artificial Biofilm and Control Test Samples

Prior to exposition, the test samples were completely covered with artificial biofilms, consisting of several layers of algal cells of intense green color, a clear signal of strong photosynthetic activity.

Following exposure to UV-C, the effects of irradiation were plainly observable: biofilms grown on the exposed test samples had become visibly bleached after the treatment, the degree of which was inversely proportional to the distance of exposition (Figure 1). The progressive bleaching of the biofilms caused by direct exposure to UV-C radiation was related to the degradation of chlorophyll, as evidenced by spectrophotometric determinations.

The exposed test samples, presented in Figure 1, showed that all microorganisms were eradicated by the lowest UV-C intensity used in this study. Higher UV-C intensities (10, 20, and 30 kJm^−2^) were sufficient to eradicate all autotrophic organisms. A selection of test samples lacking artificial biofilms, representative of each pigmentation, was then exposed to UV-C radiation. The colorimetric analysis performed on these samples showed no chromatic alteration, proving that the treatment does not interfere with the substrate.

### 3.3. Measurement of Chlorophyll Concentration

**Figure 2:**
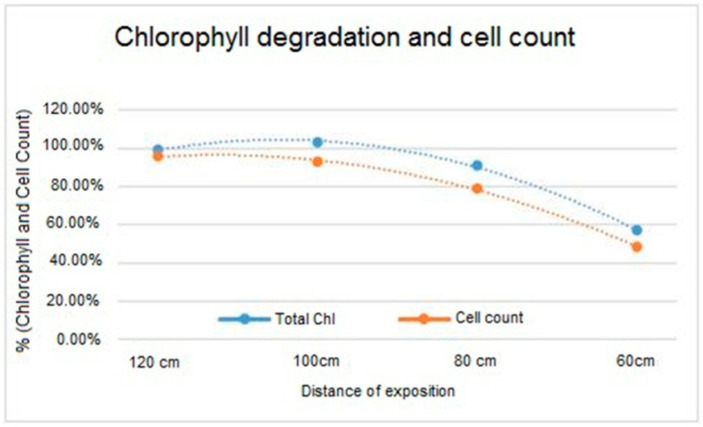
Total chlorophyll content and number of cells (expressed as a percentage of the maximum value obtained at the maximum distance of 120 cm) in samples subjected to UV-C treatment at different distances (120, 100, 80, 60 cm).

The algae exposed to the treatment showed a significant decrease in chlorophyll concentration. Furthermore, the decline was more severe in the treatment at 60 cm (with concentration firmly under 60% after irradiation), in accordance with the results of the growth inhibition test and showing a similar trend (Figure 2). Coherently with the data of the cell viability, these fluctuations were expressed as percentages.

Additionally, the concentrations of chlorophyll a and b were measured in the extended expositions (50 and 70 h), highlighting some minor differences concerning the degradation process, which shows first order kinetics.

### 3.4. Colorimetric Analyses

**Figure 3:**
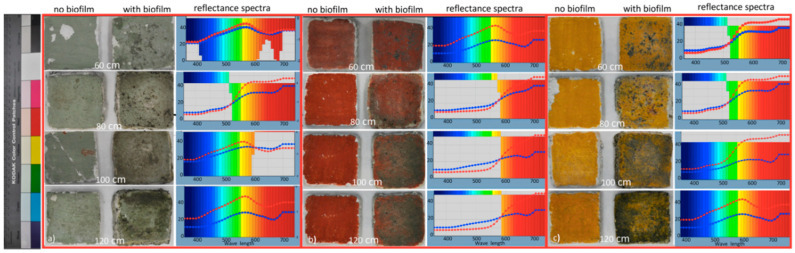
On the left side, the Kodak reference color chart is shown. In (**a**), the pictures of green color samples with and without biofilms, exposed to a different distance from UV-C irradiation are displayed. For each sample, the reflectance spectra are also reported. The red line refers to the reflectance spectrum of untreated samples exposed to UV-C radiation, while the blue line refers to samples with biofilm and subjected to UV-C treatment. In (**b**,**c**), the pictures of red and yellow samples, together with their reflectance spectra, are, respectively, shown.

In Table 1, the chromatic coordinates values of green, red, and yellow tested samples are, respectively, indicated. In Figure 3a, the photographs of green color samples with and without biofilms, exposed at a different distance from the UV-C irradiation source are shown. For each sample, the reflectance spectra are also reported. The red line refers to the reflectance spectrum of untreated samples exposed to UV-C radiation, while the blue line refers to samples with biofilm and subjected to UV-C treatment. The pictures of red and yellow samples, together with their reflectance spectra, are, respectively, shown in Figure 3b,c.

## 4. Discussion

Biodeterioration is widespread in archaeological sites as a consequence of the favorable environmental conditions [2,16,17,18]. The adaptation to such conditions allows a rapid colonization of photosynthetic microorganisms to form different surface patinas on the decorated walls; these organisms (and bacteria as well) live in a biofilm and begin their proliferation on the pigments by producing a polysaccharide matrix that in turn hosts the subsequent colonizers [1,19,20,21]. In our study, the development of photosynthetic organisms was observed on the wall paintings, especially on the red, yellow, and green pigments. We decided to choose an alternative methodology to biocides for their elimination is indeed crucial: UV-C radiation can be considered as an ideal solution to this matter. Our results shows that, under laboratory conditions, the use of UV-C light irradiation can eliminate biofilms. Whilst planning the tests described in this study, several changes to Borderie’s protocol were considered, as a means to reduce hazards related to UV-C radiation: the distance at which the treatment was carried out was increased from 30 cm to four different values, ranging from 60 to 120 cm; the exposition time was extended from under 180 min to 24 h; two 9 W lamps were used for the treatment, which represented doubtlessly a weaker, but a less dangerous, alternative to the two 50 W lamps employed previously.

The lamps used in our study proved effective at the distance of 80 and 60 cm, as they caused a substantial decline in cell viability (Figure 1 and Figure 3). In contrast, exposures at larger distances did not produce an appreciable decrease in cell count. The radiation produces visibly stronger results at shorter distances; accordingly, the exposition time should always be between 80 and 60 cm (Figure 1).

The sudden decline in survival rate is a consequence of UV-C induced damage and, especially, of the formation of cyclobutene pirimidine dimers (CPDs), which are a well-known cause of mutagenesis, senescence, and lethality of organisms cells [22,23].

In addition, UV-C radiation plays a major role in the degradation of proteins, as it was demonstrated to be especially toxic for both the structural proteins constituting the two photosystems and for some of the enzymes involved in photosynthesis [24]. The alterations caused by UV-C on photosynthetic microorganisms are described as “bleaching”, due to the evident reduction in the chromatic intensity of the pigments [25]. Spectrophotometric reads have confirmed this phenomenon, highlighting how, alongside with the decline in cell count, a significant degradation of chlorophyll had taken place. The divergent decrease kinetics of chlorophyll a and b is probably caused by the slightly different structures of the two pigments [26]. The impairment of the photosynthetic mechanism might also be related to the degradation of antioxidant enzymes, such as superoxide dismutase (SOD), and to the resultant increase in the concentration of ROS [27]. Moreover, UV-C-induced over-production of accessory pigments, such as flavonoids and anthocyanins [28], is to be considered among the mechanisms of resistance to UV-C action.

The colorimetric analysis carried out on the test samples with biofilms shows different results for the different pigments. In particular, the green samples exposed at 60 cm from the UV-C source (green 60 Figure 3a), from a macroscopic point of view, show the presence of some greyish spots even if they do not show a strong variation in both the reflectance spectrum and L*a*b* coordinates. The ∆E value is 2.54 (Table 1 green), less than 3, which represents the threshold value for appreciating color variations with the naked eye.

The green samples exposed to UV-C at a distance of 80 cm (green 80) show an increase in the blackish grey spots as well as a ∆E value up to 3.14 (Table 1 green), slightly beyond the threshold value indicated above. The reflectance spectrum, after the treatment with UV-C radiation, shows a reflectance reduction from 60 up to 45%, mainly in the wave-length region between 550 and 700 nm.

The green samples exposed to UV-C at a distance of 100 cm (green 100) show a general darkening corresponding to a 6.63 ∆E value (Table 1 green). A weak reduction in reflectance after UV-C treatment (blue line) is observed in the 400–600 nm wave length region, while a slight increase is noted in the 600–730 nm range. The shape of the reflectance curve after UV-C treatment is weakly flattened, and this corresponds to a general darkening of the surface.

The green samples exposed to UV-C at a distance of 120 cm (green 120) display a strong darkening after UV-C treatment, in good agreement with ∆L* and ∆E values, corresponding, respectively, to −12.69 and 13.27 (Table 1 green). The reflectance curve after UV-C treatment shows the same shape of that before treatment, but with a strong decrease (about 50%) of reflectance values along the entire spectrum. In addition, it is to be noted that the ∆L* value strongly increases with the distance from the UV-C source, especially for 100 and 120 green samples, and this corresponds to a high value ∆E coordinate, testifying an increase in the darkening of the surface (Figure 3a). This darkening is probably related to the death of biological communities due to the action of UV-C radiation. These data are in good agreement with those obtained by cell count determinations. 

From a macroscopic point of view:

The red samples exposed to UV-C at a distance of 60 cm (red 60) show the presence of some greyish spots, such as the green samples exposed at 60 cm, even if in this case they show a relevant increase in ∆L* value (+5.20), corresponding to a growth of ∆E value (+5.57, Table 1, red). These variations could be due to a different starting red color of the samples, also visible to naked eye. The reflectance spectrum of samples with biofilm, treated with UV-C (Figure 1b) shows the same shape of that without biofilm, but with lower reflectance values. 

The red samples exposed at 80 cm (red 80) show an increase in the blackish grey spots as well as a general darkening of all the surface corresponding to a strong decrease in ∆a (−11.96) and ∆b (−7.28) values and ∆E (14.06) as well (Table 1, red). The reflectance spectrum curve after treatment with UV-C radiation mainly shows a reflectance reduction from 45% up to 55% in the wave length region between 600 and 750 nm.

The red samples exposed to UV-C at 100 and 120 cm show a strong general darkening corresponding to high ∆E values between about 17 and 23. Both red reflectance spectra at 100 and 120 cm of UV-C-treated biofilm display the same curve (blue) showing a slight increase in reflectance in the 420–580 nm and a strong reduction in reflectance in the range between 600 and 730 nm. The shape of the reflectance curve after UV-C treatment is weakly flattened, corresponding to a general darkening of the surface, as already observed for the green samples. In these cases, the darkening is probably related to the death of biological communities as a consequence of the action of UV-C radiation. These data are in good agreement with those obtained by cell count determinations.

Referring to the red samples spectrum at 60 cm, it is to be noted that the reflectance curve, compared with those of red pigment (exposed at 80, 100, and 120 cm) shows lower reflectance values, in the range of 350–630 nm and higher reflectance values up to 750 nm. This experimental result has to be related to a different chromatic aspect of the reference sample showing, from a macroscopic point of view, a weak transparency of underlying white plaster.

Finally the yellow samples exposed to UV-C at a distance of 60 and 80 cm (yellow 60, 80) show the same strong chromatic variation due to the high presence of black-greyish spots on the surface corresponding to a high decrease in b* value (−15 and−16, respectively), which relates to a high value of ∆E (17–18, Table 1 red). In terms of L*a*b* chromatic system, the decrease in b* value means that the color has shifted to blue.

The reflectance spectra curves of the yellow samples exposed at 60 and 80 cm show the same behaviour, confirming the above displayed data.

The yellow samples at 100 cm (yellow 100) show a strong darkening of surface corresponding to a high decrease in all L*a*b* chromatic coordinates that in turn implies a high value of ∆E (21,82, Table 1). In terms of reflectance spectra (Figure 3c), the samples with UV-C-treated biofilm display a strong decrease in reflectance values in the wavelength between 500 and 750 nm. 

From a macroscopic point of view, the yellow samples exposed at a distance of 120 cm (yellow 120), compared to the yellow 100 cm distance samples, display a higher level of darkening, not corresponding to a further decrease in the L*a*b* (expected) values of chromatic coordinates (Table 1).

In terms of reflectance spectrum, the samples with UV-C-treated biofilm show a decrease in reflectance value over all wavelength range. As observed for the green and red samples, the general increase in darkening means that the UV-C radiation does not simply stop the growth of biological communities but causes their death after exposition, confirming the results obtained from cell count measurements.

The colorimetric analyses also yielded information about the effects of UV-C radiation on the pictorial layers of samples without biofilm. In this case, the curves of the untreated green samples placed at 60, 80, 100, and 120 cm from the UV-C source show the same behaviour with some slight deviations one from each other, testifying that the UV-C treatment is not invasive in terms of color changes of surfaces.

For the red and yellow samples, the same behaviour of reflectance spectra (red line) has been observed.

Only for the red samples at 60 cm, and for the yellow samples at 120 cm, a different behaviour of the reflectance curve (red line) is observed, very likely linked to a transparency effect of the painted surface, already present before the application of UV-C radiation.

## 5. Conclusions

In order to control biodeterioration, knowledge of the typologies of biofilm is necessary to establish adequate restoration strategies. Methods and products must be selected according to the conditions of the substrate and to the species to be eradicated. The chemical compounds normally used to inhibit the proliferation of stone colonizing microorganisms, although harmless for the surfaces of interest, may pose a serious threat to the surrounding ecosystems. In fact, these biocides are characterized by a long durability, which, together with the phenomenon known as “lixiviation” (i.e., water running on stone surfaces extracting these compounds and depositing them into water ways), makes them harmful for the environment [7].

In light of all the above, we believe that this study provides suggestions for a safer and more effective employment of UV-C irradiation as an environmentally friendly replacement for ecotoxic biocides. This will be of the utmost importance for archaeological sites characterized by open or semi-confined spaces in natural surroundings.

Starting with a specific conservation problem, that is, preservation of mural paintings in very wet environments (in this case a semi-hypogeal tomb), we found a possible protocol of application of the UV-C irradiation, which uses its biocidal action.

The first experimentations of this method were carried out in 1995, both on mural paintings [29] and on mosaics [30,31], but ultimately disregarded until several years later when subsequent experimentations were carried out by French research teams on specimens collected from the lampenflora of Dordogne caves [3,5,7,21].

Even if electrical power supply may represent a problem in some cases, alternative solutions can be easily found, such as batteries, solar energy systems, etc.

The 8 h cycle fits especially well within the daily rhythm of a touristic location, such as the necropolis of Porta Nocera, in Pompeii; this treatment may in fact take place mostly at night, so as to mitigate possible human hazard. It may also be safely adapted to treat different biodeterioration at different degrees: the use of slightly more powerful lamps (30 W) may represent an ideal step forward in this respect. This protocol will, therefore, prove optimal to keep the costs low and minimize collateral damage, which can be caused by the repeated application of chemicals in very wet environments.

The experiments carried out in this work verified the compatibility of the UV-C irradiation method with inorganic pigments; we also verified that heating of the surfaces during the treatment is not relevant. Irradiation showed biocidal action also in presence of a complex colonization. Therefore, the method proposed here may be regarded as a valid alternative to the need of repeated application of biocides.

Furthermore, the possible application of the method to prevent the biological growth, as an implementation of the UV-C rays method, could be safer both for the artwork but also for the environment and the operator.

A further implementation of this protocol may take into account the possibility to find the optimal conditions (type of lamp and exposure distance) to avoid re-growth of biodeteriogens and subsequent chemical cleaning.

## Figures and Tables

**Table 1:** Colorimetric data.

Sample	∆L*	∆a*	∆b*	∆E
Green				
green 60 cm	−2.40	1.10	0.01	2.64
green 80 cm	−1.40	2.70	0.35	3.14
green 100 cm	−3.51	5.23	2.08	6.63
green 120 cm	−12.69	1.22	3.68	13.27
Red				
red 60 cm	5.20	−1.47	−1.38	5.57
red 80 cm	1.31	−11.96	−7.28	14.06
red 100 cm	3.38	−21.30	−9.5	23.56
red 120 cm	1.33	−17.15	−4.89	17.88
Yellow				
yellow 60 cm	−5.20	−5.69	−15.01	16.95
yellow 80 cm	−6.15	−6.09	−16.23	18.39
yellow 100 cm	−15.38	−9.09	−12.87	21.82
yellow 120 cm	−12.69	−1.22	−3.68	13.27
